# The Effects of Heavy Metal Pollution on Soil Nitrogen Transformation and Rice Volatile Organic Compounds under Different Water Management Practices

**DOI:** 10.3390/plants13060871

**Published:** 2024-03-18

**Authors:** Muhammad Afzal, Sajid Muhammad, Dedong Tan, Sidra Kaleem, Arif Ali Khattak, Xiaolin Wang, Xiaoyuan Chen, Liangfang Ma, Jingzhi Mo, Niaz Muhammad, Mehmood Jan, Zhiyuan Tan

**Affiliations:** 1 College of Agriculture, South China Agricultural University, Guangzhou 510642, China; mafzal.kust@gmail.com (M.A.); arifalikh2020@hotmail.com (A.A.K.); xlwang@scau.edu.cn (X.W.); 11514056@zju.edu.cn (L.M.);; 2 Guangdong Provincial Key Laboratory of Utilization and Conservation of Food and Medicinal Resources in Northern Region, Shaoguan University, Shaoguan 512005, China; chenxy2@163.com; 3 College of Agriculture and Biotechnology, Zhejiang University, Hangzhou 310058, China; msajid1772@163.com; 4 School of Resources Environment and Safety Engineering, University of South China, Hengyang 421001, China; sehrishcherry127@gmail.com; 5 Riphah Institute of Pharmaceutical Sciences, Islamabad 44600, Pakistan; sidra.kaleem@riphah.edu.pk; 6 Department of Microbiology, Kohat University of Science and Technology, Kohat 26000, Pakistan; mniaz@kust.edu.pk

**Keywords:** cadmium, rice, N cycle, paddy soil, 2-acetyl-1-pyrroline, isoprene

## Abstract

One of the most concerning global environmental issues is the pollution of agricultural soils by heavy metals (HMs), especially cadmium, which not only affects human health through Cd-containing foods but also impacts the quality of rice. The soil’s nitrification and denitrification processes, coupled with the release of volatile organic compounds by plants, raise substantial concerns. In this review, we summarize the recent literature related to the deleterious effects of Cd on both soil processes related to the N cycle and rice quality, particularly aroma, in different water management practices. Under both continuous flooding (CF) and alternate wetting and drying (AWD) conditions, cadmium has been observed to reduce both the nitrification and denitrification processes. The adverse effects are more pronounced in alternate wetting and drying (AWD) as compared to continuous flooding (CF). Similarly, the alteration in rice aroma is more significant in AWD than in CF. The precise modulation of volatile organic compounds (VOCs) by Cd remains unclear based on the available literature. Nevertheless, HM accumulation is higher in AWD conditions compared to CF, leading to a detrimental impact on volatile organic compounds (VOCs). The literature concludes that AWD practices should be avoided in Cd-contaminated fields to decrease accumulation and maintain the quality of the rice. In the future, rhizospheric engineering and plant biotechnology can be used to decrease the transport of HMs from the soil to the plant’s edible parts.

## 1. Introduction

Rice serves as a staple food for half of the global population, with numerous Asian and African nations, such as Madagascar and Liberia, exhibiting annual per capita rice consumption exceeding 100 kg. In Africa, the consumption of rice is increasing more rapidly as compared to other commodities because of the growing urban population [1,2]. In 2023, about 523.1 million tons of rice were produced globally [2]. The majority of rice production comes from Asian countries, with significant contributions from Indonesia, Vietnam, Thailand, Myanmar, China, Bangladesh, India, the Philippines, and Pakistan, as reported by the Food and Agriculture Organization (FAO) in 2023. Western and Eastern Asia together account for over 90% of both global rice production and consumption. China’s role in worldwide rice production stands at 41% [3]. This accounts for approximately 23% of China’s total cultivated land [3,4]. However, a total of 150 million acres of China’s arable land is contaminated with heavy metals (HMs), accounting for 8.7% of total arable land [5]. Of the HMs, Cd has polluted more than 12,000 ha in 11 irrigated regions of China [6].

Water is a crucial element for the growth and development of rice plants. One widely used rice irrigation method around the globe is continuous flooding (CF). However, CF results in unproductive water loss such as seepage, evaporation, and percolation [7,8,9]. To overcome unproductive water loss and maintain a high rice yield, water-saving irrigation methods have recently been introduced. However, a few long-term field studies have been conducted, which provide a little information about crop yield during water-saving practices in the fields [7]. Because freshwater is distributed unevenly in terms of both space and time, farmers strive to conserve as much rainwater as they can. Farmers utilize this water for irrigating rice fields when the soil reaches a specific dryness threshold. Currently, the following rice irrigation modes are in use throughout China: continuous flooding, midseason flooding with intermittent drying, midseason flooding with intermittent irrigation, and midseason flooding with rain-fed conditions. The most popular method is flooding–midseason drying–flooding [7].

Nitrogen (N) cycling involves both biotic and abiotic transformations of the nitrogen in paddy soils. These transformations encompass processes such as ammonification, N immobilization, nitrification, denitrification, anaerobic ammonium oxidation (anammox), dissimilatory nitrate reduction to ammonium (DNRA), and nitrogen fixation. In paddy soils, the importance of nitrification and denitrification are well documented and a recent addition to this is archaeal ammonium oxidation [10,11,12]. Reports indicate that in ammonia oxidization to nitrite, carried out by microorganisms encoding the enzymes of ammonia monooxygenase (AMO) [13], ammonia oxygenation serves as the initial rate-limiting step in nitrification. The AMO enzyme has been reported in ammonia-oxidizing bacteria (AOB) and/or ammonia-oxidizing archaea (AOA) [14,15,16]. Nitrifier microorganisms are distributed in few functional taxa, while denitrifying bacteria are broadly distributed in many taxa that are responsible for the nitrate as an alternative electron acceptor for respiration [17].

Plants interact with plants and other living communities. Therefore, they have developed a variety of strategies. One of them is the diffusion of volatile organic compounds (VOCs); these signals provoke a defense mechanism against biotic and abiotic stresses and several other environmental factors [17]. Angiosperm and gymnosperms release a number of VOCs, such as fatty acids, amino acid derivatives, phenylpropanoids/benenoids, and terpenoids, which are most common [18,19,20]. Through priming neighbors, plants develop stress resistance due to the VOCs of stress-exposed plants [21]. This phenomenon was established between tobacco (*Nicotiana attenuate*) and sagebrush (*Artemisia tridentata*) plants [22]. The former was exposed to the VOCs of the latter clipped one. The clipped sagebrush mimicked insect damage and the tobacco developed herbivore resistance.

Soil acts as a seed bank of microbes, and plant roots recruit the microbes from the soil. The presence of heavy metals in the soil leads to changes in both the soil biomass and the microbial community structure. In the rhizosphere of contaminated plants, roots exhibit a higher prevalence of heavy metal-resistant bacteria but a lower overall microbial population compared to the rhizosphere of unpolluted plants [23,24,25,26]. Within the soil, cadmium [1] induces alterations in the microbial community structure, reduces microbial carbon use efficiency, and results in an increase in the microbial C:N ratio [27]. Recent studies in metal-polluted soil have focused on the microbiome of model plants such as *Arabidopsis thaliana*, metal-accumulating or metal-tolerant and hyper-accumulating plants [28,29,30]. Both biotic and abiotic factors shape microbial community assembly in the plant rhizosphere [31]. The ecology of the rice microbial community under metal stress, particularly Cd stress, is the least studied area. The majority of metal-contaminated soil-grown plant rhizospheres consist of metal-resistant bacteria that can maintain metabolic functions [32,33]. *Thiobacillus*, *Pedobacter*, and *Geobacter* have been reported as high metal-resistant bacteria genera responsible for Fe (III) reduction in the Cd-contaminated rice rhizosphere [34,35,36]. However, some plant growth-promoting rhizobacteria (PGPR) such as *Dyella* and *Novosphingobium* colonized the rice rhizosphere [36]. Furthermore, the role of VOCs in soil microbial N transformation also needs to be discussed in detail during HM stress.

Nitrification, denitrification, VOCs, and plant endophytes are very important factors in terms of plant health. In this review, we will focus on Cd’s detrimental effects on the N transformation, the VOCs of rice, and endophytes under different water management practices.

## 2. Different Water Management Practices for Rice Cultivation

Globally, about 4 billion people are suffering from water scarcity [37]. Furthermore, for sustainable rice production, a shortage of water is a major threat. In order to ensure food security, alternative approaches such as using less water for rice cultivation are very important. Therefore, in modern agriculture practices, water-saving irrigation for rice cultivation must be the focus of concentration in future research. Various approaches and technologies can be taken into account, such as alternate wetting and drying (AWD), reducing the outflow of unproductive water, adopting direct seeding, implementing the rice intensification system, employing the rice aerobic system, embracing the ground cover rice production system, and utilizing tensiometers for scheduled irrigation. These technologies can improve water use efficiency and significantly decrease the demand for water for the production of rice.

### 2.1. Continuous Flooding (CF)

Warm and waterlogged soil is used for rice cultivation. Continuous flooding (CF) is a traditional rice-growing practice in which the rice paddies are flooded by the farmers throughout the rice-growing season. Globally, 70% of rice cultivation is carried out under CF. CF-based rice cultivation is highly productive but some problems related to soil health and the environment have been reported. Soil degradation, elevated methane emissions, and the substantial accumulation of harmful substances like arsenic and mercury are primarily associated with rice cultivation using chemical fertilizer-based practices [38].

Lowland rice irrigation covers about 79 million hectares worldwide, with an estimated 75% of the world’s rice production. The global crop irrigation area covered in Asia is approximately 56%, where 40–46% is rice cultivation [39]. About 64–83% of the irrigated area in Southeast Asia, 46–52% in East Asia, and 30–35% in South Asia are occupied by rice cultivation. In comparison to other irrigated crops in the field, rice needs 2–3 times more water [40]. Of the developed 70% freshwater resources worldwide, rice irrigation receives a 24–30% share.

Several factors, such as global warming, rice competition with other seasonal crops, industries, and civic requirements for water, may cause economic or physical water shortages for rice cultivation [41]. In the future, several million hectares of lowland rice irrigated systems will face water shortages by 2050 [7]. On account of all the issues related to the CF rice irrigation system, the development of water-saving irrigation systems is compulsory for sustainable rice cultivation [7]. The International Rice Research Institute (IRRI) introduced alternate wetting and drying (AWD) water management technology, among other water-saving rice irrigation systems, to deal with the expected water shortages in the future. AWD has attracted worldwide attention and several research studies are available that are becoming increasingly widespread [42].

### 2.2. Alternate Wetting and Drying

Developing agronomic practices that minimize water usage for irrigation while sustaining high yields is essential to support the increasing global population. To address the issue of water scarcity in rice agricultural production, alternate wetting and drying (AWD) water management practices are being adopted [38,43]. Under AWD conditions, rice fields are drained for a certain period in which the field obtains a certain level of moisture and are then re-flooded. It has been reported that using AWD water technology saves about 23% of the water used for rice irrigation when compared to CF practices [8,40]. AWD technology has the potential to reduce greenhouse gas (GHG) emissions, particularly methane (CH_4_) gas [38,44]. Alternate wetting and drying conditions decreased global warming potential (GWP − CH_4_ + N_2_O) by about 45–90% compared to CF. Human activities generate about 48 Gt CO_2_ annually, which can increase global warming. Therefore, to reduce GHG emissions from paddy fields, it is critical to promote AWD adaptation globally [45]. In the USA, for example, farmers received carbon credits under the project “Voluntary Emission Reductions in Rice Management Systems”, which involves farmers adopting practices aimed at minimizing greenhouse gas (GHG) emissions, with AWD being one such practice [46]. On the other hand, AWD also reduced arsenic (As) accumulation in rice grains [43,47] and methylmercury concentration in soils, and lowered the consumption of energy/fuel where pumping is in practice for irrigation [38,48,49]. Many efforts have been made to promote AWD in Asia because of its benefits [50]. In China, the “midseason” drain is widely adopted, which is somewhat similar to AWD [51]. However, global AWD technology adoption is limited due to a variety of factors limiting rice productivity [38,48,52].

Since the 1990s, China has embraced water-saving irrigation methods such as thin–shallow–wet–dry irrigation [53] and alternate drying–wetting [54,55]. Soil water management strategies influence oxygen transfer, nitrogen transformation, and soil redox potential [11,12,56]. Soil microbial biomass carbon (MCB) and nitrogen (MBN) have been significantly affected by irrigation practices [57,58]. In paddy soils, MBC and MBN decreased in continuous flooding irrigation as compared to intermitted irrigation [59]. Other studies have reported high N_2_O emissions from paddy fields with intermittent irrigation and midseason drainage [60,61].

Soil moisture is very important during the rice grain filling stage. Grain quality and yield are both impacted by water levels during rice reproduction and the filling stage [62,63]. Both CF and AWD effectively enhanced the quality of rice grains [64]. For example, AWD improved grain head recovery, grain weight, and protein content [65,66]. Similarly, rice kernel opaqueness, abortiveness, and chalkiness were reduced significantly in AWD [62,66]. In addition, AWD significantly increased the nutritional value of the rice grain such as high antioxidants, total tocopherols, γ-oryzanol, flavonoids, zinc, and iron [67]. Furthermore, AWD significantly decreased arsenic in the rice grain [68].

### 2.3. Popular Methods around the Globe

Globally, rice production and greenhouse gas production are under the influence of two important factors, i.e., water management and the application of nitrogen fertilizer. China is one of the largest rice-producing countries; water shortages threaten rice cultivation. The most popular rice cultivation method is CF. However, due to water shortages and greenhouse gas emissions, nowadays different water-saving irrigation systems are in practice around the globe (Table 1). Among the different water-saving irrigation methods, AWD is the most practiced technology for rice production.

However, different studies have reported that AWD decreased CH_4_ and increased N_2_O emissions. Moreover, while applying medium nitrogen (N), the optimal rice grain yield has been observed in the case of AWD in contrast to the conventional flooding–midseason drainage–flooding irrigation (PFD) approach. Different studies from Bangladesh, the Philippines, China, and Pakistan reported a 4.52% average increase in rice yield (t/ha) under AWD as compared to CF. Similarly, water use efficiency (WUE) also increased in AWD as compared to CF.

Another rice production system, termed the aerobic rice system (ARS) [48], is applied in different regions of the world. In the ARS, rice is grown under non-saturated, non-flooded, and non-puddled soil conditions [69]. The yield obtained from the ARS was higher than in upland conditions but lower than in flooded conditions. In cool temperate regions, the ARS is a successful system for rice production. However, the partial aerobic rice system (PARS) is more suitable alternative than the ARS in warm humid regions. There have been reports that the ARS reduces rice yields over time, and it has been proposed that this technique may not be ideal for long-term rice agriculture [70]. Several factors, including Zn, Fe, K, N, and P deficiencies, root-knot nematodes, and weeds are responsible for the yield reduction in the ARS [71]. However, it saves about 50% of irrigation water compared to CF.

**Table 1:** Different strategies used for water-saving irrigation of rice production.

S. No	Technologies for Conserving Water in Rice Cultivation	Reference
1	Surface irrigation techniques in rice cultivation.	[72,73,74]
	2. Drip irrigation methodology in rice cultivation.	[70]
	3. Sprinkler irrigation approach for rice cultivation.	[75]
2	Alternate wetting and drying (AWD)	[12,76]
	2. Soil water potential (SWP)	[38,77]
	3. Mulching cultivation without flooding	[78,79]
	4. Aerobic rice system	[48]
	5. Optimal irrigation regimen (OIR)	[75]
	6. Saturated soil culture (SSC)	[80]
	7. Field water level (FWL)	[75]
	8. Intermittent drainage (ID)	[81]
	9. Leaching and flushing methods (LFM)	[75]
	10. Conventional flooding–midseason drainage–flooding irrigation (FDF)	[1]

### 2.4. Heavy Metal Pollution in Paddy Soil

Cadmium [1] is considered one of the most hazardous heavy metals, which permeates the soil through various sources [11]. Its pollution is a severe global issue since it can build in agricultural soil and edible sections of crops, and eating those contaminated components can cause cancer in humans [12]. Its various natural and anthropogenic sources include rock weathering, atmospheric deposition, urbanization, wastewater irrigation, sewage sludge application, the overuse of phosphate fertilization, mining, and smelting-originated Cd contamination in the soil [82]. Geological parent material decides the background Cd level. However, it has generally been reported as being in the range from 0.06–1.1 mg kg^−1^ with a 0.01 mg kg^−1^ lower value and 2.7 mg kg^−1^ higher value [82]. However, due to human activities, a higher bioavailable Cd level (up to 14 mg kg^−1^) has been reported in several regions of the world [11,12]. These studies extracted the Cd content via 0.01 M CaCl_2_ and determined it by ICP-MS. Several factors such as the cultivar, crop species, and soil properties contribute to the accumulation of Cd in plants.

### 2.5. Sources

#### 2.5.1. Natural Means of Cd Origination (Geogenic)

Naturally, Cd exists in all rocks and all soils. Among the top 20 priority hazardous metals, Cd is ranked 7th [83]. Different rocks have different Cd concentrations. For example, sedimentary deposits rocks have higher Cd levels than igneous and metamorphic rocks [84,85,86]. Where soils develop on phosphorites, shales, or carbonates, then the geogenic soil Cd concentration ranges from 0.01 to 10 or 100 mg kg^−1^ [87,88]. Cd exhibits weak sorption to the surfaces of both organic matter and minerals in the soil. Cadmium from mineral lattices is released slowly by the weathering of high-Cd parent rocks [89].

It is hard to distinguish between soil Cd of geogenic or anthropogenic origin. Generally, Cd-enriched topsoil is assumed to occur via farm and atmosphere inputs. Nevertheless, this is not the case, as Cd is transported to subsoils through the biological cycle [90]. Furthermore, it is transported via vegetation, as Cd in agriculture is brought from subsoils to the topsoil [91]. At a continental scale, the concentration of Cd in European soil is primarily influenced by geological factors rather than anthropogenic inputs, despite the widespread use of intensive fertilizers, urban waste, and manures in agriculture [92].

#### 2.5.2. Anthropogenic Sources of Cd Input

Human activities are the leading cause of worldwide Cd deposition in the environment, accounting for 13,000 tons of the 30,000 tons of Cd released to the environment each year [12,93]. In agricultural soils, Cd is introduced by various sources and its concentration is generally lower than 0.2 mg kg^−1^. The level of Cd in agricultural soils has increased due to long-term phosphate fertilizer application, along with sewage water used for irrigation purposes [94]. Discharged wastewater, mining activities (exhaust and solid waste), the smelting sector, and atmospheric deposition all also contribute to Cd contamination of agricultural soil. Blast furnace sludge and flue dust contain a relatively high content of Cd [95]. The oxidizable and residual fraction of textile dyeing plant sludge has been reported to consist of an average 3.72 mg kg^−1^ Cd [96]. In Changsha City, which is the capital of the Hunan Province of China, dust analysis showed that 50% of the Cd was insoluble and reducible fractions in PM_2.5_ (particulate matter with aerodynamic diameters equal to or less than 2.5 µm) from the re-suspension of dust, fuel combustion, vehicular emissions, and other pollution sources were based on speciation analysis during spring [97]. Nevertheless, the predominant source of Cd input into the soil arises from agricultural activities, such as the irrigation with wastewater, unnecessary phosphate and organic fertilizer applications, and the random disposal of untreated urban sewage sludge and garbage [98]. The issue of Cd input via phosphate fertilizer application in the agricultural soils of China is controllable because phosphate and Cd content are naturally low in the native phosphate rocks of China, which leads to imports of high-quality phosphate fertilizers that contain a high level of Cd [99]. The Cd content was about 7.5–156 mg kg^−1^, 84–144 mg kg^−1^, 9.5 mg kg^−1^, and 24.5 mg kg^−1^ in imported ammonium phosphate, single superphosphate, normal superphosphate, and triple-superphosphate, respectively, which exceeded the standards [99]. The atmospheric deposition of Cd in paddy soil has been reported to be about 65–70% [100]. Similarly, fertilizers, particularly phosphate fertilizers and biosolids, contribute 10–50% each to Cd accumulation in paddy fields [101]. However, in China, animal manure contributed about 55% to total soil Cd inputs [102].

### 2.6. Chinese Paddy Fields and Heavy Metals (HMs)

In China, paddy soil Cd contamination is a widespread problem. The Cd paddy soil issue is more severe in some mining and industrial regions of China. Paddy soil Cd contamination ranges from 0.01 to 5.50 mg kg^−1^, with an average value of 0.23 mg kg^−1^ [103]. Hunan has been reported to have the highest Cd (0.73 mg kg^−1^), whereas Guangxi and Sichuan provinces have been recorded as having 0.70 and 0.46 mg kg^−1^ of paddy soil Cd. However, in the 1990s, the background value of the Cd in Chinese paddy soil was 0.1 mg kg^−1^ [100]. Paddy Cd contamination across China has followed a trend from Northwest > South central > East > Northeast > North [100]. In China, paddy fields are mostly contaminated by Cd due to the smelting of the ores of Cu, Pb, and zinc [93,104]. The Cd content of rice grains varies greatly. Rice grains sampled from fields and markets have been reported to have raised levels of Cd of up to 7.0 and 1.24 mg kg^−1^, respectively [104]. A study based on 100 rice grain samples in Hunan, one of the large nonferrous metal production zones, reported only 15% of rice grains met the standard level for As, Cd, and Pb [105]. Another study randomly collected 63 rice grain samples from the open markets of Jiangxi and Anhui, along with 7 samples from Guangdong., Hunan, and Jiangxi, and the Cd level in about 70% of the grain samples was found to be higher than the standard 0.2 mg kg^−1^ [106].

## 3. HMs and Soil N Transformation

Rice plants prefer ammonium to nitrate. When both ammonium and nitrate are present, rice seedlings exhibit a faster uptake of NH_4_^+^ compared to NO_3_^−^ [107]. In the past half-century, due to the NH_4_^+^ preference of rice, farmers used to cultivate rice in paddy fields where ammonium-based fertilizers like ammonium sulfate and urea were applied [107]. In addition, organic fertilizers for rice cultivation are also commonly applied in the fields, in which the organic N is converted to ammonium through a biological process called ammonification (mineralization of N). Due to the low level of oxygen or depletion in paddy soils, the rate of ammonification is slower compared with upland soils [108]. The depletion of O_2_ in soils not only slows down the ammonification but also limits the process of ammonium oxidation (NH_4_^+^ to NO_3_^−^) and the immobilization of N (microbial assimilation of NH_4_^+^, i.e., the conversion of inorganic N to organic N), which leads to the accumulation of NH_4_^+^ in soils [109].

### 3.1. The Significance of Nitrification and Denitrification Processes in Paddy Soils

The most limiting nutrient for rice production is generally considered to be N. Denitrification in paddy soils was initially studied in order to make efficient use of fertilizers and link gaseous products such as NO, N_2_O, and N_2_ with the microbial process involved in the reduction of nitrate [110]. The thin oxidized layer of paddy soils is the region where nitrification occurs, while denitrification takes place below the oxidized layer [111]. The loss of N from paddy soils includes ammonia volatilization, nitrate leaching, and nitrification [112]. Denitrification occurs just after nitrification in paddy soils. It is proposed that ammonium fertilizers should be deeply applied before waterlogging (4–5 days) in paddy fields to overcome the N loss from the paddies [113]. Studies on N transformation on a microscale in the flooded soil surface layer reported a higher level of nitrifying and denitrifying bacteria in the top 2 cm layer than in the lower reduced layer of paddy soil [13,114]. After one to two weeks of waterlogging, low molecular weight organic molecules (acetate) accumulated in the top layer, which increased the denitrifying bacteria. The correlation of organic carbon and denitrifying bacteria in other environments, including lake sediments and river waters, also have been reported [115]. The most important factor for the controlling of denitrification is the availability of organic C since it provides electron donors for the denitrifiers. Rice plant roots release O_2_ in the rhizosphere; therefore, nitrification and denitrification activities were also reported for the rice rhizosphere [116,117] and the green algae that grow in rice fields [118], as well as in the bulk soil between the oxidized and reduced layers. The process of nitrification–denitrification in rice paddy soil may cause the loss of N from the applied fertilizer. Globally, 1.3–1.8% of the total greenhouse gas emissions are recorded from paddy soils. Different studies have reported lower emissions of N_2_O from paddy soil under controlled condition than from upland field crops [119,120]. Denitrification is a strong activity in rice paddy soils; as a result, little emission of N_2_O occurs by the conversion of N_2_O into N_2_ [121]. Furthermore, several studies on dissolved N_2_O on the rice surface and in the groundwater have been conducted [122]. These findings suggest that little emission of N_2_O gas from rice paddy soil occurs, due to the N_2_O reducers present in the rice paddy fields [123]. However, recent findings suggested that iron plaque in the rhizosphere contributes considerably to N_2_O emissions, which was previously ignored [124].

### 3.2. Nitrification

**Figure 1:**
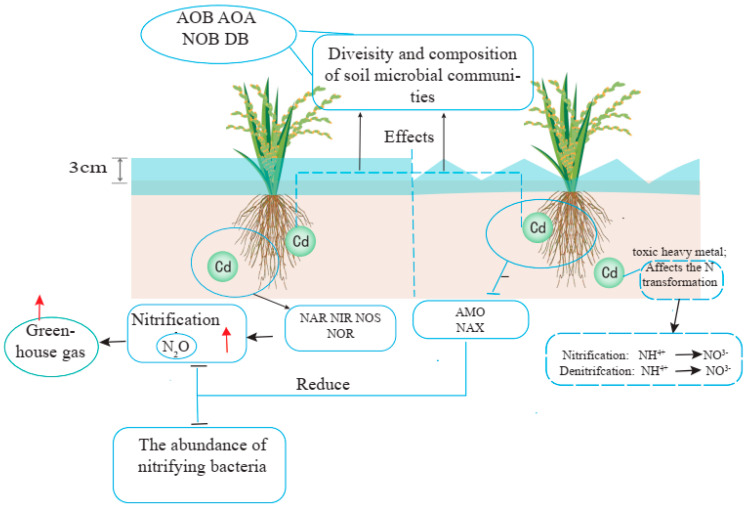
Paddy soil N transformation under different water management practices and Cd contamination. Note; The red color upward arrows shows gas emission, the black color arrows left or right, up or down shows the transformation or the flow of processes. ↕ Shows the water level above the soil surface.

Nitrification is a totally microbial-mediated process in which nitrate (NO_3_^−^) formation takes place by ammonium (NH_4_^+^) oxidation via nitrite (NO_2_^−^) [11,125,126] (Figure 1). This is the energy-acquiring process for autotrophic nitrifiers. The process in which NO_3_^−^ or NO_2_^−^ formation takes place from inorganic N (NH_4_^+^) and organic N without energy acquired by microbes is known as heterotrophic nitrification and the microbes are termed heterotrophs [125].

Nitrification has been divided into two steps: the oxidation of ammonia (NH_4_^+^→NO_2_^−^) and nitrite oxidation (NO_2_^−^→NO_3_^−^). Regarding the *Nitrosospira* spp. and *Nitrosomonas* spp. of *Betaproteobacteria* and the *Nitrosococcus* spp. of *Gamma proteobacteria*, these bacteria are known as ammonia-oxidizing bacteria (AOB) [13] and can carry out the oxidation of ammonia. Archaea, along with bacteria, can also develop in ammonia oxidation, a process termed ammonia-oxidizing archaea (AOA) [14,127,128]. Different studies have reported a higher number of AOA than AOB in the ocean [129]. A positive correlation of AOB abundance was observed with rice paddy nitrification activity in the soil, which indicates that AOB could be the major players in nitrification, rather than AOA [130]. However, the application of urea fertilization increased the abundance of both AOA and AOB in the rice rhizosphere [131,132]. Recent reports have shown the relative contribution of comammox to nitrification is higher than AOA and AOB in rice paddy soil [130].

The diversity of ammonia-oxidizing bacteria [11] communities has been investigated in rice paddy soils [133]. The rice genotype can also influence the diversity of AOB [134]. In Chinese rice paddy soil, the *Nitrosospira* spp. are the most dominant AOB [135,136,137], while biochar-amended rice paddy soil favored the *amoA* gene containing AOB related only to the *Nitrosomonas* spp. [138]. Among other factors, fertilizer practices may change the AOB community in the soil, because in environments with high ammonia concentrations (like wastewater), the *Nitrosomonas* spp. predominate, while in the soil the *Nitrosospira* spp. is the major AOB [139]. Nitrite-oxidizing bacteria (NOB) from the genera of *Nitrobacter*, *Nitrospina*, *Nitrococcus*, and *Nitrospira* perform nitrite oxidation, which is the second step of nitrification [140,141]. In our previous studies, we have observed Cd inhibit nitrification by decreasing the abundance of nitrifying microbial communities [11,112]. In addition, the bioavailable portion of the Cd was higher in AWD than in CF, which decreased the AOA and AOB microbial community significantly higher than CF. Therefore, nitrification was significantly decreased in AWD as compared to CF in paddy soil.

### 3.3. Denitrification

Denitrification is a process in which a gaseous form of N (NO, N_2_O, and N_2_) forms via the stepwise reduction of NO_3_^−^ and NO_2_^−^ by microbial respiration (Figure 1). Microbes utilize nitrogen oxides as electron acceptors in lieu of oxygen when operating under anaerobic conditions for respiration.

Nitrous oxide also acts as an electron acceptor but non-denitrifiers reduce N_2_O [142]. Two types of nitrite reductases are responsible for denitrification, i.e., copper-containing NirK encoded by the gene *nirK*, and the NirS cytochrome cd1-containing enzyme encoded by the *nirS* gene [14]. These enzymes transform oxidized ammonia into NO and N_2_O. Finally, the nitrous oxide reductase enzymes encoded by *nosZ* reduce N_2_O into N_2_. The nitrite reductases (*nirK* and *nirS*) gene-related community has been studied in detail [14,143]. In rice paddy soil, the average gene copy numbers of *nirK*, *nirS*, and *nosZ* were 7.65 × 10^−7^, 3 × 10^−7^, and 8.71 × 10^−7^, respectively [11], and 16S sequencing approaches in paddy soil revealed that denitrifier community genes are distributed in *Betaprotobacteria*. The phylum was further divided into *Azospira* and *Burkholderiales*, belonging to the genus *Herbaspirillum*, and *Rhodocyclales* in rice paddy soil under denitrifying conditions [144].

The soil type, plant genome, pH, Eh, organic matter, temperature, substrate, and fertilizers affect the response of the microbes to denitrification. For example, increasing the temperature increases the denitrification rate in paddy soil [113,145]. Similarly, high moisture also increases denitrification [113]. However, the opposite is true for N fertilizer. N fertilizers increase the *nirK* gene abundance and decrease *nirS* in paddy fields [143].

#### 3.3.1. Response of Nitrifier Microbial Communities to HMs in Paddy Soil

The physical and chemical properties of soil influence the microbial-mediated process rates in field environments [146,147,148]. The nitrification and denitrification processes are not only affected by the soil pH but also, as a recent study reported, by soil NH_4_^+^-N and C/N, which can also be responsible while changing the abundance of soil microbial communities [149]. The effect of metals on ammonia oxidation depends upon the specific metal or combination of metals in the environment; e.g., no change in the abundance of AOA was reported in mercury-spiked soil used for vegetable cultivation, while Cu-spiked soil at a rate of up to 1000 mg kg^−1^ changed the AOA community in grassland and arable soils [150,151]. A study has reported that Cu was more toxic to the ammonia oxidizer community as compared to Ni. For example, Cu strongly affects the AOB community in sludge-amended soil and adaptability was more common to Zn and Ni [152]. However, the general microbial community was also shown to be more vulnerable to Cu and Cr contamination as compared to Zn and Ni [153]. Heavy metal pollution had a negative effect on the abundance, diversity, activity, and composition of AOA and AOB in natural soil [12]. Another study reported a higher copy number of AOA than AOB in rice paddy fields contaminated with heavy metals and the ratio of AOA to AOB copy numbers was in the range of 2.5 to 59 [151]. In our previous studies, we have reported that high Cd availability was present in AWD as compared to CF [11,12]. Similarly, AOA and AOB communities were significantly decreased in AWD as compared to CF. Therefore, nitrification decreased more significantly in AWD than in CF.

#### 3.3.2. Response Denitrifies Microbial Community to HMs in Paddy Soil

Denitrification is a heavy-metal-sensitive process and the heavy metal concentration in the soil is directly proportional to its inhibition [154]. Heavy metals change the denitrifiers in the soil, which reflects the inhibition of denitrification (Figure 1). A recent study reported a significant change in the composition of the denitrifiers in a metal-polluted paddy soil via denaturing gradient gel electrophoresis (DGGE) [155]. In addition, in rice paddy soil, the microbial community has been reported to adapt to metal stress by changing the abundance of certain microbial taxa. Not only the abundance but also the functional potential has been reported to be altered in metal-polluted paddy soil. The replacement process is the main factor in the adaptability to heavy metals of the microorganisms, while soil pH variation is totally responsible for the community richness [156]. The negative effect of metals on the abundance of denitrifiers also varies with the elements. The copy of the *nirK* and *nosZ* genes sharply decreases with spiked Cu in a 6-day and Cd in a 56-day laboratory incubation experiment, but a recovery in copy numbers of denitrifying genes has been reported for long incubation [1,157]. Heavy metal pollution, especially with Cd, decreases the abundance, diversity, and structure of both nitrifiers and denitrifiers in rice paddy soil [111,158]. Similarly, the high bioavailable portion of heavy metals in paddy soil in AWD is more detrimental to denitrifers than in CF [11,12]. Overall, HM decreases denitrification more in AWD than in CF.

#### 3.3.3. Greenhouse Gases from Paddy Fields under Metal Contamination

The application of N fertilizers is essential for rice production with a high yield. However, due to the overuse of N fertilizers, the production of greenhouse gases (GHGs) such as nitrous oxide (N_2_O) is one of the major concerns [159,160]. Different studies have addressed these problems by specifying which soil types are more prone to N losses by describing many environmental and management factors linked to biological nitrogen transformation [161,162]. However, the question of how heavy metal pollution can affect the biological transformation in the soil is still to be answered, especially regarding the introduction of heavy metals through atmospheric deposition versus metals spiked into the soil, which suddenly increases the heavy metal concentration in the soil [152,163]. Another issue is the link between changes in N transformation gene abundance and heavy metal contamination in soils. It will become more difficult to address this issue in rice paddy soils where significant changes occur in the redox potential due to wet/dry cycles that affect the heavy metal bioavailability, the structures of the microbial community, and the rate of the biological transformations of N [156,164].

Among all the GHGs, N_2_O is the most radiative GHG, increasing 0.26% in the atmosphere per year [156]. Incomplete denitrification results in the production of nitrous oxide in which the last reduction step fails to reduce the N_2_O into N_2_ in the soil. It has been reported that about 60% of the total N_2_O globally is produced by anthropogenic sources, with the major contributors known to be paddy fields [165,166]. China’s paddy fields produce up to 29.0 Gg of N_2_O emissions, which represent about 7–11% of total GHG emission from China’s croplands [53,167]. Recent studies have shown that in metal-polluted paddy soils, the microbial biomass and fungal-to-bacterial ratio declined with an increase in the concentration of heavy metals, which could lead to changes in carbon (C) cycling. Heavy metal contamination in soil could affect the rates of microbial-mediated biogeochemical processes. For example, soil nitrification rates were suppressed by metal salts such as CdCl_2_ and ZnCl_2_, both in spiked soil samples in short-term studies and in polluted fields where metals typically accumulated at a slower rate [168,169,170,171]. In a study using surface wetland sediments spiked with multiple metals, total denitrification activity was inhibited by Cd (30.9%), Zn (24.9%), and Cu (18.9%) over a period of 7 days of incubation, with Cd being the strongest inhibitor [172]. Heavy metal pollution was found to affect the activities of denitrifying bacteria and ammonia oxidizers in soil [173]. In our previous study, we reported lower N_2_O production in AWD than in CF [11]. The reason for this lower emission is possibly the decreased abundance of the *nosZ* gene in AWD.

### 3.4. Different Forms of Cd and Its Mineralization under Changes in Redox Conditions of the Soil

Various forms of heavy metals are consistently found in the soil, each exhibiting distinct levels of toxicity, bioavailability, and solubility such as (1) being insolubly precipitated with other soil, (2) having structural elements of lattices within clay minerals, (3) being substitutable in both organic and inorganic components, and (4) being dissolved (in soil solution [174]. The properties of soil such as pH, soil redox potential (Eh), and cation exchange capacity, and organic matter content, e.g., Mn and Fe oxides, clay minerals, and calcium carbonate, influence HM speciation and availability in the soil [9]. Changes in soil Eh impact the bioavailability of Cd due to alterations in redox status and variations in electron acceptors. In aerobic oxidizing conditions, Cd commonly exists as a soluble salt and cationic form, specifically Cd^2+^ However, in anaerobic situations with a low redox potential, Cd tends to exist as precipitated species like cadmium sulphate (CdS) and cadmium carbonate (CdCO_3_) [175]. Conversely, alterations in redox potential influence both the organic matter and mineral components of the soil [176]. Furthermore, soil redox conditions regulate the microbial community characteristics and functions [177,178].

The soil moisture regime regulates both soil Eh and biological activity. Paddy soil typically experiences saturated conditions. Heavy metals are found in solid-phase components, and the introduction of heavy metals to paddy soil leads to their transition from more labile fractions to less labile fractions [179,180]. At field capacity moisture, Cd exhibits lower stability when compared to saturated conditions [181]. The saturated regime results in a more complete transformation of metals toward stable fractions than the field capacity regime [181]. Soil kept dry exhibits significantly higher soluble concentrations of Cd, Cu, and Zn than the field capacity and the saturated treatment [182].

Management practices such as air drying cycles are pertinent to soil moisture conditions, influencing the exchangeable fraction of heavy metals in the soil. Air drying enhanced Cd desorption in soil and the leachability of metals from dredged canal sediment increased during drying and oxidation processes [183,184]. Drying the soil in an oven resulted in notable rises in the concentrations of dissolved copper (Cu) and nickel (Ni). This outcome can be attributed to the complexation of these metals with increased levels of humic acid and fulvic acid in the soil [185]. Air drying enhanced the extractability of manganese [120], iron (Fe), copper (Cu), and zinc [93] at low incubation moisture levels, while reducing the extractability of metals at high incubation moisture levels [186]. In paddy soil incubated under three distinct moisture regimes, the exchangeable fraction of copper (Cu), lead (Pb), and cadmium [1] exhibited the following order: 75% field capacity > wetting–drying cycle > flooding. Conversely, the reducible fraction followed the reverse order [187,188]. Eh stands as a crucial factor influencing the mobility of metals in soil or sediment, and is closely tied to soil moisture regulation [189]. Under oxidizing conditions, there is a discernible likelihood of significant heavy metal release [190]. High Eh is more related to the high availability of HM than to anoxic conditions in paddy soil.

## 4. Toxic Effects of Cd on Rice

Elevated cadmium [1] concentrations have the potential to impede plant growth and reduce grain yield and quality [190]. This can result in modifications to plant tissues and disrupt the process of photosynthesis [190], and regulate the expression of at least 36 proteins in rice [191]. The accumulation and transport of Cd occur in various organs and cell organelles once it enters into the rice [192]. Different researchers have reported the toxic effects of Cd on plant growth [193,194]. Stunted growth and chlorosis are the most typical Cd toxicity symptoms [195]. Due to the direct and indirect interaction of the high level of Cd in the leaves, chlorosis (chlorophyll loss) takes place. The change in physiological activities of plant-like respiration, cell proliferation, and photosynthesis, indirectly [195], and nitrogen metabolism, the plant–water relationship, and mineral nutrition under direct Cd stress have been reported, which resulted in poor growth and a low biomass of the plants [196]. Sensitivity to drought, enzyme inactivation, and damage to the membranes of plants are well-established facts of Cd toxicity [197]. It indirectly stops the photosynthesis of the plant by closing the stomata due to water conductance, which reduces the access of CO_2_ to the plant [198]. Directly, Cd prevents photosynthesis by affecting electron transport, chloroplast organization, chlorophyll biosynthesis, and the activity of the Calvin–Benson–Bassham cycle enzymes [199,200].

The general symptoms of Cd toxicity in rice plants include growth inhibition, chlorosis of the leaves and sheaths, brown root systems, and wilted leaves [180,201]. Different metal–sulfide complexes make a brown pigment that is deposited on the surface of rice roots [202]. The production of lipid peroxides is a response to Cd toxicity in rice plants. In addition, the uptake of plant essential elements such as Fe, Zn, Mn, and Cu has also been reported to be affected by Cd toxicity in rice plants [203]. In addition, the light-harvesting center and photosystem II, along with chlorophyll metabolism in rice leaves, are affected by Cd [202].

## 5. HMs and VOCs in Paddy Soil under Water Management Practices (DWMP)

**Figure 2:**
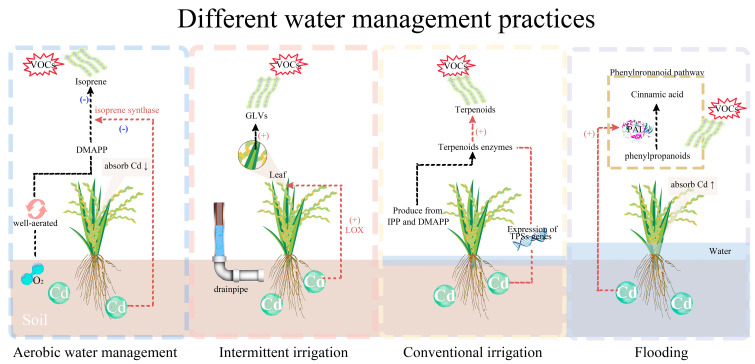
Effects of DWMP on rice VOCs under Cd contamination. Note: (−) stop a process or emission, (+) start the production or emission, ↓ arrows shows decrease in production or accumulation and ↑ shows increase in production or accumulation.

Various water management practices influence the Cd uptake in rice (Figure 2). Volatile organic compounds (VOCs) emitted by vegetation encompass a wide array of chemical molecules, predominantly comprising isoprene, monoterpenes, and methanol [17,204]. In any case, the variety of plant-emitted VOCs depends on the plant species, the distinctive parts of the plants, and the growth conditions [205]. Plants can emit higher quantities of these molecules by directing a greater amount of carbon into stress-triggered volatile organic compounds (VOCs) when subjected to attacks by pathogens or herbivores. Additionally, exposure to various abiotic stresses such as ultra-violet (UV) radiation, ozone, drought, eutrophication, warming, and overall oxidative stress can also contribute to the increased release of these compounds [206,207,208,209]. In fact, many VOCs of the family of terpenes, such as isoprene and monoterpenes, are antioxidants and can protect cells from oxidative stress [210,211].

Under the aerobic irrigation of rice, Cd uptake and isoprene emission decreased [212,213]. Isoprene (2-methyl-1, 3-butadiene) is an important plant VOC known for its plant protection role against numerous abiotic stimuli, including heat [213] and drought stress [214]. Cd decreases the activity of isoprene synthase, an enzyme responsible for the conversion of dimethylallyl diphosphate (DMAPP) to isoprene [215,216]. Similarly, one class of VOCs that Cd amplifies is green leaf volatiles (GLVs), which play a part in plant defense [215,216]. Furthermore, Cd contamination modulates the emission of terpenoids and the expression of genes encoding terpene synthases (TPSs). TPSs are responsible for different terpenoid formation utilizing isopentenyl diphosphate (IPP) and DMAPP [217].

### Effects of Heavy Metals on Rice Fragrance

**Figure 3:**
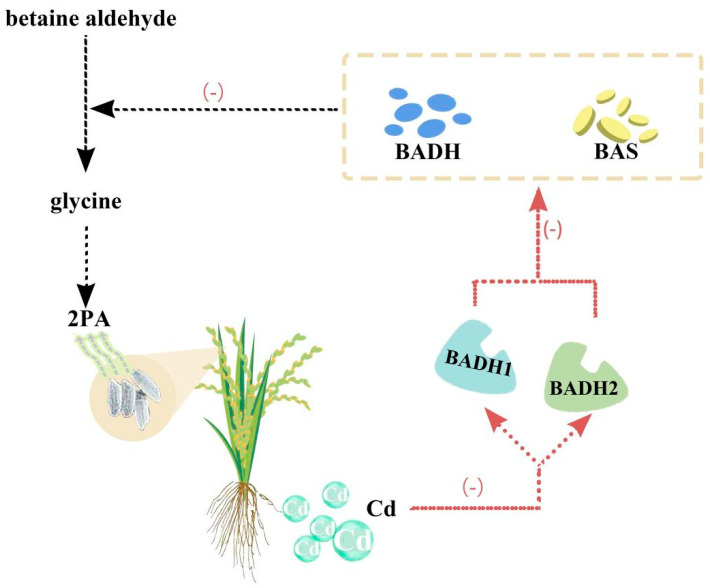
The effect of Cd on rice genes responsible for fragrance.

Aromatic 2-acetyl-1-pyrroline is the main ingredient in rice fragrance (2AP). Two genes, betaine aldehyde dehydrogenase (*BADH1*) and *BADH2*, function in the 2AP biosynthetic pathway. The exact mechanism by which Cd affects these genes is not well studied. However, studies have reported that Cd may interfere with the expression and functions of *BADH1* and *BADH2*, limiting rice yield and quality. For example, upon exposure to Cd, the expression of *BADH1* and *BADH2* is reduced in rice seedlings. Similarly, Cd lowered the activity of enzymes encoded by these *BADH* genes and betaine aldehyde synthase (Figure 3) [28,218]. These enzymes are responsible for converting the betaine aldehyde into glycine, a precursor of 2AP. Glycine is a compatible solute that protects plants from stress. The decreased activity of these enzymes lowers the level of glycine betaine and raises the level of betaine aldehyde, leading to plant toxicity. Furthermore, the accumulation of 2PA accumulation due to Cd in the grain also affects the rice fragrance [219].

The duplication of the *BADH2* gene in rice decreased Cd accumulation in the rice roots and shoots under salt and Cd stress. Duplication increased the expression and activity of *BADH2*, which takes up more manganese, a micronutrient and competitor with Cd for transport in rice cells [219]. Furthermore, the introduction of the *BADH2* gene duplication into various rice cultivars reduced Cd accumulation without impacting yield or grain quality [219]. Similarly, 96 rice accessions were found to have 12 single nucleotide polymorphisms (SNPs) in six cadmium-related genes, comprising *BADH1* and *BADH2* [101]. SNPs are linked with Cd accretion in rice grains. In rice-breeding practices, SNPs might be used as molecular markers for low-Cd-accumulating rice cultivars.

## 6. Conclusions and Future Directions

The high bioavailability of Cd under AWD conditions decreases nitrification, denitrification, and GHG emissions. Similarly, higher Cd accumulation is related to its bioavailability, which is higher in AWD as compared to CF. The defining factor for paddy soil Cd bioavailability is the Eh of the soil. More oxidized conditions favor Cd bioavailability. Therefore, due to the high bioavailability of Cd, rice aroma and grain quality are more compromised in AWD as compared to CF. Higher Cd accumulation in grains raises food security and human health-related issues. Similarly, Cd interferes with the signaling and synthesis of plant cells that are responsible for VOC production and aroma. A high Cd deposition in rice cells inhibits VOC formation; nevertheless, the precise mechanism by which VOC synthesis is modulated by DWMP remains unknown. This may compromise both plant defenses that are induced by VOCs and rice aroma. The issues of rice aroma, quality, and yield can be mitigated in future breeding procedures by introducing low Cd absorption rice cultivars and utilizing water-saving technology.

Currently, Cd accumulation in grains is reduced via agronomic parameters (soil improvement, water, fertilizer, tillage management) and applying silicon and abscisic acid externally. Another strategy is microbial and phytoremediation. However, these processes are cost-ineffective and are laborious with low efficiency. Therefore, screening and breeding low-Cd-accumulating rice varieties is the only choice considered economical and effective. Crossbreeding is very effective in transferring a low Cd gene pool to offspring. However, molecular marker assistant breeding can reduce the screening process in breeding and save time. In addition, mutation breeding also helps to develop low-Cd-accumulating varieties quickly. However, molecular marker assistant breeding can reduce the screening process in breeding and save time. Clustered Regularly Interspaced Short Palindromic Repeats (CRISPR) and hybridization technologies can also be used to develop new low- Cd-accumulating rice varieties. Adaptations of such techniques by breeders may develop rice varieties that can be grown in Cd-polluted paddies but will maintain their aroma and grain quality.

## Data Availability

No new data were created or analyzed in this study. Data sharing is not applicable to this article.
